# Transcriptomic profiling of germinating seeds under cold stress and characterization of the cold-tolerant gene *LTG5* in rice

**DOI:** 10.1186/s12870-020-02569-z

**Published:** 2020-08-06

**Authors:** Yinghua Pan, Haifu Liang, Lijun Gao, Gaoxing Dai, Weiwei Chen, Xinghai Yang, Dongjin Qing, Ju Gao, Hao Wu, Juan Huang, Weiyong Zhou, Chengcui Huang, Yuntao Liang, Guofu Deng

**Affiliations:** 1 Rice Research Institute, Guangxi Academy of Agricultural Sciences/Guangxi Key Laboratory of Rice Genetics and Breeding, Nanning, China; 2 Guangxi Academy of Agricultural Sciences/Guangxi Crop Genetic Improvement and Biotechnology Laboratory, Nanning, China

**Keywords:** Wild rice, RNA-Seq, *LTG5*, Cold tolerance

## Abstract

**Background:**

Low temperature is a limiting factor of rice productivity and geographical distribution. Wild rice (*Oryza rufipogon* Griff.) is an important germplasm resource for rice improvement. It has superior tolerance to many abiotic stresses, including cold stress, but little is known about the mechanism underlying its resistance to cold.

**Results:**

This study elucidated the molecular genetic mechanisms of wild rice in tolerating low temperature. Comprehensive transcriptome profiles of two rice genotypes (cold-sensitive ce 253 and cold-tolerant Y12–4) at the germinating stage under cold stress were comparatively analyzed. A total of 42.44–68.71 million readings were obtained, resulting in the alignment of 29,128 and 30,131 genes in genotypes 253 and Y12–4, respectively. Many common and differentially expressed genes (DEGs) were analyzed in the cold-sensitive and cold-tolerant genotypes. Results showed more upregulated DEGs in the cold-tolerant genotype than in the cold-sensitive genotype at four stages under cold stress. Gene ontology enrichment analyses based on cellular process, metabolic process, response stimulus, membrane part, and catalytic activity indicated more upregulated genes than downregulated ones in the cold-tolerant genotype than in the cold-sensitive genotype. Quantitative real-time polymerase chain reaction was performed on seven randomly selected DEGs to confirm the RNA Sequencing (RNA-seq) data. These genes showed similar expression patterns corresponding with the RNA-Seq method. Weighted gene co-expression network analysis (WGCNA) revealed Y12–4 showed more positive genes than 253 under cold stress. We also explored the cold tolerance gene *LTG5* (Low Temperature Growth 5) encoding a UDP-glucosyltransferase. The overexpression of the *LTG5* gene conferred cold tolerance to indica rice.

**Conclusion:**

Gene resources related to cold stress from wild rice can be valuable for improving the cold tolerance of crops.

## Background

Rice is an important staple crop for food production worldwide. Rice grains are consumed by nearly half of the world’s population [1]. An increase in rice yield and production has become an important issue concerning the global economy and food security due to rapid population growth and the decrease in arable land. Rice is sensitive to low temperature (LT) that originated from tropical or sub-tropical regions [2, 3]. LT is a major limiting factor in rice production worldwide [4]. LT can cause chlorosis, necrosis, and growth retardation at germination. Cold stress induced reactive oxygen species (ROS) and malondialdehyde levels increase, electrolyte leakage, changes of proline content, rigidifies the membrane, destabilizes protein complexes, tissue browning and impairs photosynthesis which result in damages such as reduction in germination and vigor, delay in seedling emergence, delay in initial growth, and low percentage [5, 6]. In China, annual losses of 3–5 million (M) metric tons are due to LTs [7]. The wild relative *Oryza rufipogon* is the closest to cultivated rice. *O. rufipogon* has a broad geographical distribution with various characteristics resistant to biotic and abiotic stresses for adapting to different ecological and agronomic conditions [8]. As such, wild rice can supply abundant genetic diversity resource to provide a valuable gene pool for rice genetic improvement. Cold-tolerant wild rice can be a good genetic source in developing cold-tolerant rice cultivars.

RNA-Seq technology and digital gene expression are highly efficient methods of identifying cold-tolerant genes in rice under abiotic stress by analyzing expression profiles, single-nucleotide polymorphisms (SNPs), and alternative splicing [9–11]. Transcriptome resources have several applications, such as in functional genomics of walnuts, building an optimal gene coexpression network of maize, studying flowering and shade-avoidance pathways in wheat, and analyzing transcriptomic changes in *Pinus koraiensis* under cold stress [12–15]. Transcriptome analysis in two indica rice genotypes (cold tolerant and cold sensitive) revealed that the biological processes enhanced in cold-tolerant seedlings include membrane transport capacity, sucrose synthesis, hormone, and Ca^2+^ signaling. Meanwhile, the synthesis of heat shock proteins and dehydrins responds to LT stress in cold-sensitive seedlings [16]. Comparison of the transcriptomes of genotypes Oro (tolerant rice) and Tio Taka (sensitive rice) showed that cold treatment influences the expression of genes involved in the metabolic routes of signal transduction, phytohormones, antioxidant system, and biotic stress [9]. A transcriptome analysis (RNA-seq) in three cold-tolerant genotypes and one cold-sensitive genotype showed that the calcium signal transduction and RNA helicases play a dominating role in the cold stress response of rice [17]. RNA-seq technology has been used to evaluate the whole-genome transcriptome of cultivated rice (Kongyu 131 and 9311) and weedy rice (WR 03–35 and WR 03–26) under cold stress [18]. Transcriptome analysis using gene expression profile in chilling-tolerant and chilling-sensitive genotypes revealed that multiple regulatory pathways work together under LTs in rice [19].

In the present study, we used the Illumina sequencing platform to investigate a cold-tolerant wild rice (*Oryza rufipogon* Griff.) and a cold-sensitive indica rice genotype at LT (4 °C) at the germination stage. The differentially expressed genes (DEGs) in the common wild rice and indica rice were screened and identified. A gene ontology (GO) enrichment analysis was performed to identify and characterize the biological processes involved in the response of rice to cold stress. Over the past two decades, many studies have revealed that cold stress responses in rice involve complex regulatory networks [20, 21]. The cold tolerance (CT) of rice at different growth stages is controlled by different genes [22]. A few genes conferring CT have been identified, such as *COLD1*, *qLTG3–1*, *LTG1*, *Ctb1*, *CTB4a*, and *LTT7* [23]. The chilling tolerance divergence1 is a plasma membrane-localized protein that can sense cold signals, and the COLD1–RGA1 complex mediates the cold-induced influx of intracellular Ca^2+^, leading to the activation of COR genes [23]. *qLTG3–1* encodes an unknown protein and tightly associates with the vacuolation of tissues covering the embryo to improve CT in rice at the germination stage [24]. *LTG1* encodes a casein kinase and plays an important role in adaptive growth [25]. The F-box protein gene *Ctb1* confers CT that is associated with greater anther length [26]. *CTB4a* (CT at the booting stage) encodes a conserved leucine-rich repeat receptor-like kinase and improves CT in crop plants at the booting stage [10]. *LTT7* is a protein gene that provides strong tolerance to LT at the early seedling stage in rice [27]. In terms of molecular mechanism, comprehensive physiological and metabolic routes are involved in the response of rice at LT. Cold signaling pathways determine the CT of plants. LT stimulates membrane fluidity, and membrane protein conformation that causes cold signal is transmitted in the intracellular region [28, 29]. The membrane and temperature sensor (COLD1/RGA1) receives the cold signal, leading to an influx of Ca^2+^, ROS production, abscisic acid accumulation, and mitogen-activated protein kinase (MAPK) cascade (*OsMKK6*-*OsMPK3*) reactions [21, 30, 31]. ICE-CBF-COR is one of the most widely reported pathways [32]. CBF orthologs have been isolated in rice [21]. Histone acetylation of *OsDREB1b* in rice is induced by LT [33]. *OsMYBS3* suppresses the *DREB1*-dependent pathway under prolonged cold stress in rice [34]. Ca^2+^-dependent kinase24 (*OsCPK24*) can regulate rice to confer cold tolerance by causing the phosphorylation of glutathione-dependent thiol transferase Grx10 [35]. *OsMPK3* enhances *OsICE1* protein stability by phosphorylating *OsICE1* and disrupting its interaction with the E3 ubiquitin ligase O*sHOS1*, thereby improving the chilling tolerance of rice [36]. Despite these studies, the molecular mechanisms underlying CT at the germination stage remain unclear because of the complexity of the associated genetic pathways.

These analyses illustrate many cold stress-responsive genes that are present in wild rice. However, the molecular basis of cold-tolerance mechanisms in wild rice remains unclear. This study showed that the wild rice gene *LTG5* played a role in the high tolerance of this crop to LT. The mechanisms of CT or cold adaptation were compared among wild rice, indica, and *LTG5* to explore CT genes and thus improve the breeding of cold-tolerant rice. The results can contribute to our understanding of the cold-response mechanisms in rice and wild rice and provide technological resources for improving biotechnology and molecular breeding in rice.

## Results

### Phenotypic differences between the cold-sensitive line ce 253 and cold-tolerant wild rice Y12–4 under cold stress

**Fig. 1:**
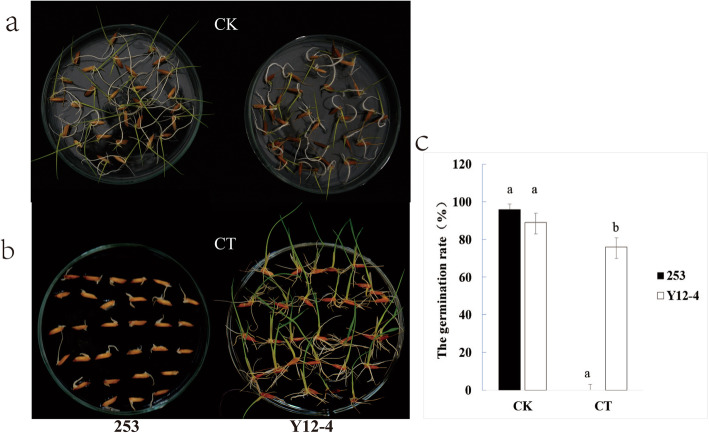
The comparison of the germination rate under low temperature of 253 and Y12–4 in the control and in the cold treatment. CK indicates control treatment; CT indicates cold treatment. Vertical bar indicates standard error. **a**: 253 and Y12–4 under 25 °C for 7d; **b**: 253 and Y12–4 under 4 °C for 10d and recovered for 5d; **c**: the germination rate of 253 and Y12–4 under normal and cold treatment

The cold-sensitive indica 253 and common wild rice (*O. rufipogon* Griff.) Y12–4 possessing a CT phenotype were used in this study. The two genotypes were obtained from Guilin, Guangxi Province, China. Under cold stress, the seeds were prepared with a coleoptile length of ≥5 mm. A marked difference in survival rate was observed between 253 and Y12–4 (Fig. 1), with Y12–4 showing better CT and recovery ability than 253. The germination rate of Y12–4 was 76% and that of 253 was 0% under 4 °C for 10 days and recovered for 5 days (mean values were compared by Student’s t test, *P* ≤ 0.01) (Fig. 1).

### Library construction and sequencing

**Fig. 2:**
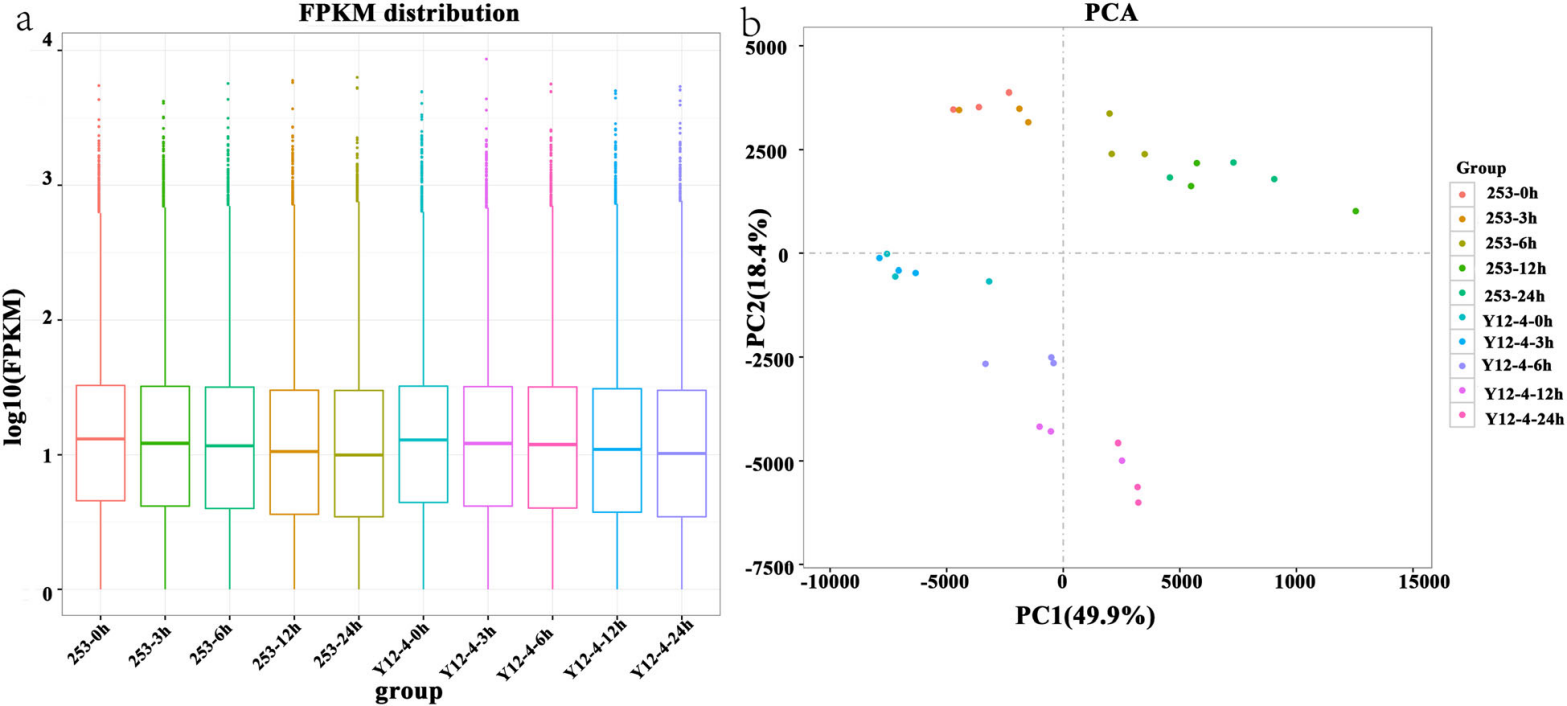
FPKM distribution and PCA of rice sampled

Clean reads were collected from each sample, and a limited transcriptome that provides a profile of the transcript dynamics under cold stress was obtained. Cold (4 °C) conditions influenced gene expression and induced different kinds of rice at the global transcriptome level (Fig. 2a), indicating that cold induced an extensive activation of transcription. Moreover, principal component analysis revealed a different activation of gene networks involved in cold-induced responses between 253 and Y12–4 (Fig. 2b). PC1 (49.9%) and PC2 (18.4%) explained 68.3% degrees in 30 variables. Correlation analysis indicated large differences among the sample treatments (Supplementary Figure 1).

**Fig. 3:**
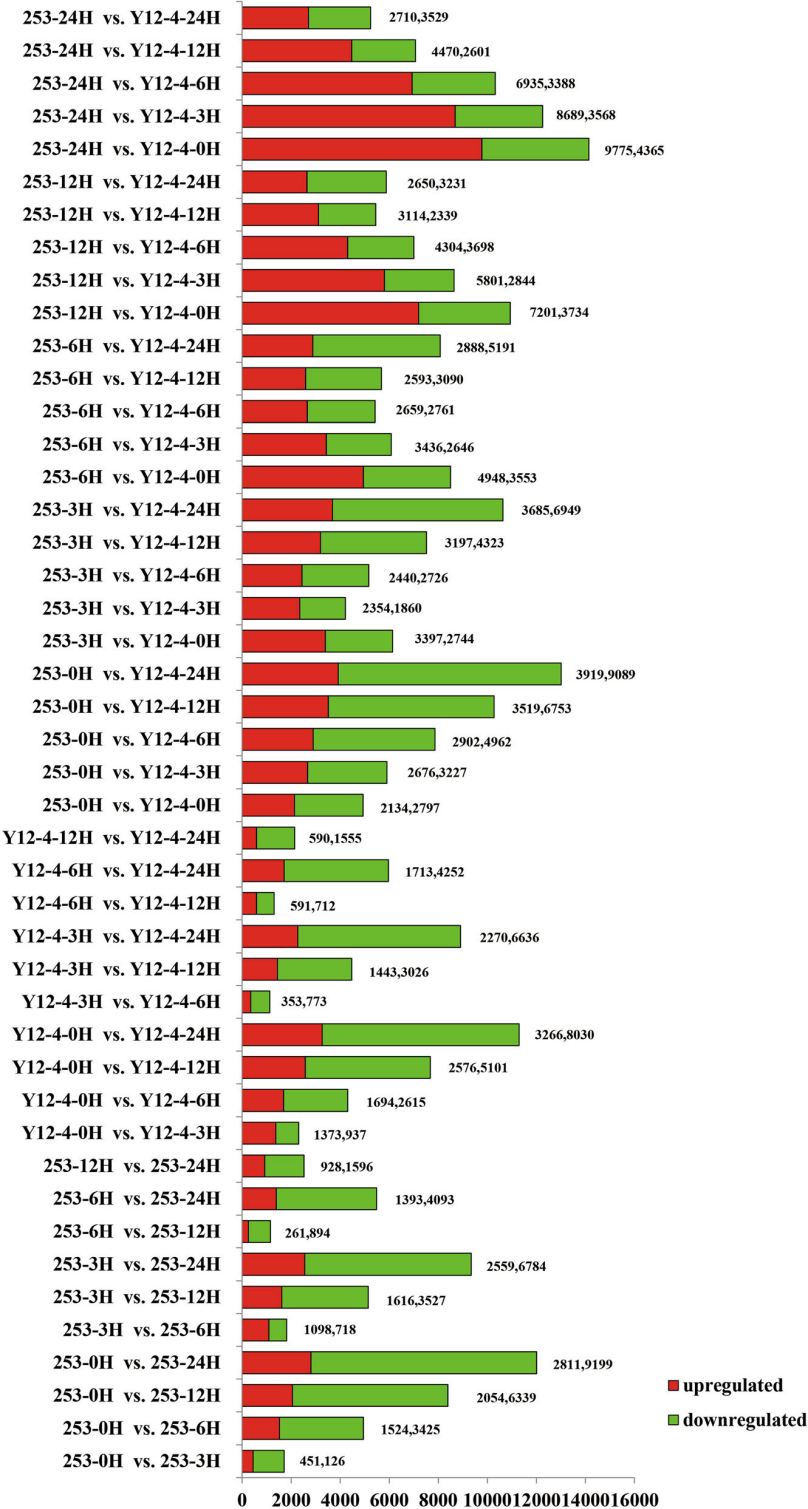
Comparison of the differential expressed genes of each pairs. The labels of the samples are displayed as “hours after cold treatment” (H)

**Fig. 4:**
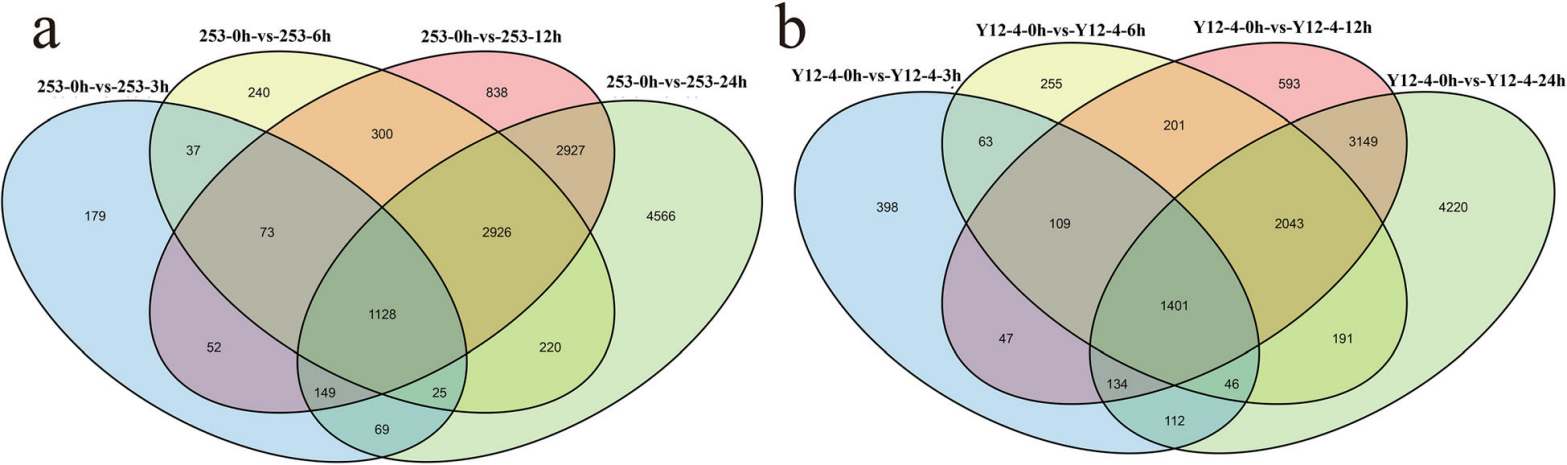
Venn map of the differentially expressed genes in the two rice genotypes under low temperature. **a**: Venn map showing the expressed genes of different cold responsive genes in 253 genotype. **b**: Venn map showing the expressed genes of different cold responsive genes in Y12–4 genotype

High-throughput sequencing generated 42.44–68.71 M 100 bp paired-end reads from each library (Table 1). A total of 31,577 genes were defined as known genes (84.53% of the total model 37,358 genes). After filtering out new transcripts shorter than 200 bp and those with only one exon, 2238 transcripts were predicted as new genes. RNA-Seq results showed that 577 (451 upregulated/126 downregulated), 4949 (1524 upregulated/3425 downregulated), 8393 (2054 upregulated/6339 downregulated), and 12,010 (2811 upregulated/9199 downregulated) DEGs were found in rice buds after 3, 6, 12, and 24 h in 253 under the same cold conditions, whereas 2310 (1373 upregulated/937 downregulated), 4309 (1694 upregulated/2615 downregulated), 7677 (2576 upregulated/5101 downregulated), and 11,296 (3266 upregulated/8030 downregulated) DEGs were found in rice buds after 3, 6, 12, and 24 h in Y12–4 (Fig. 3). Notably, more upregulated genes were found in cold-tolerant Y12–4 than in cold-sensitive 253 at 3, 6, 12, and 24 h. Y12–4 had fewer downregulated DEGs than upregulated DEGs after 3 h than at other stages. The results indicated that more genes were upregulated actively in response to LT stress in Y12–4. The 253 and Y12–4 genotypes at the germination stage were subjected to cold (4 °C) for 0, 3, 6, 12, and 24 h. Cold treatment affected the global patterns of gene expression. Under cold stress, 253 and Y12–4 showed 1128 and 1401 differentially expressed transcripts, respectively (Fig. 4a and b).

**Table 1:** Statistics on the quality and output of the RNA-Seq libraries

Classification	Maxinum	Mininum	Average
No.raw reads	68,713,906	42,446,108	54,010,614.30
No.clean reads	67,557,942	41,624,368	53,065,008.70
No.mapped reads	54,813,859	31,960,338	42,558,059.20
% of mapped reads	85.97%	77.18%	83.29%
No.perfect mapped reads	54,006,323	31,575,048	41,939,296.60
% of perfect mapped reads	84.75%	76.28%	82.09%
Q30%	95.83%	94.52%	94.92%
Total base pair	1,591,950,262		
coverage of the transcriptome	243,047,764,500

**Fig. 5:**
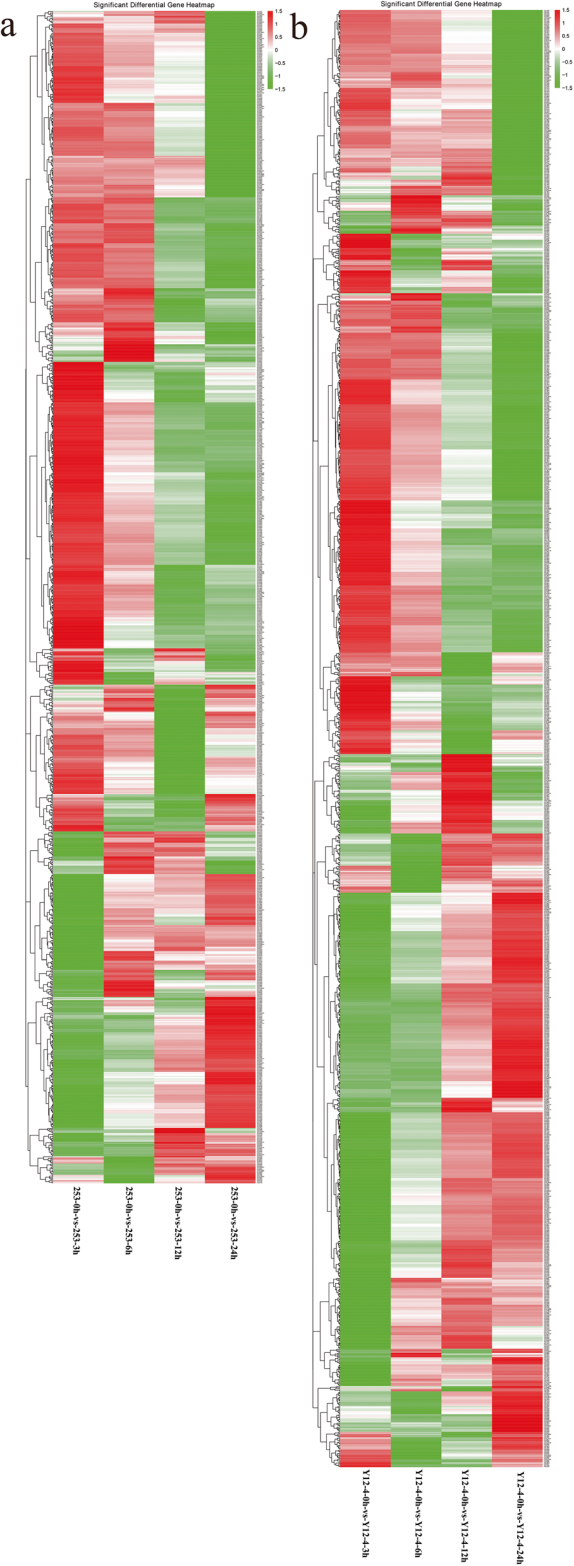
Hierarchical clustering of the DEGs for the two rice lines under the cold treatment. **a**: Heat map showing the expression patterns of different cold responsive genes in 253 genotype. **b**: Heat map showing the expression patterns of different cold responsive genes in Y12–4 genotype

To identify the gene expression patterns, we performed hierarchical clustering of DEGs for the two rice lines under cold treatment at 0, 3, 6, 12, and 24 h according to the log2 (fold change) values > 1 and corrected *P*-value < 0.05 (Fig. 5). RNA-Seq data analysis revealed 4566 and 4220 DEGs in 253 and Y12–4, respectively (Tables S1 and S2). The complex transcriptional response pattern to early cold stress in 253 and Y12–4 can be visualized by hierarchical clustering. In addition to the remarkably increased number of upregulated and downregulated genes after cold treatment, the expression ratio also changed considerably between 253 and Y12–4 relative to the control, as indicated by the heatmap analysis (Fig. 5). Long-term exposure (24 h) to cold stress increased the number of regulated genes. Few genes supported the basic mechanism in both varieties, but several distinct genes responded to LT in 253 and Y12–4.

### Co-expression network analysis for identification of cold-related DEGs

**Fig. 6:**
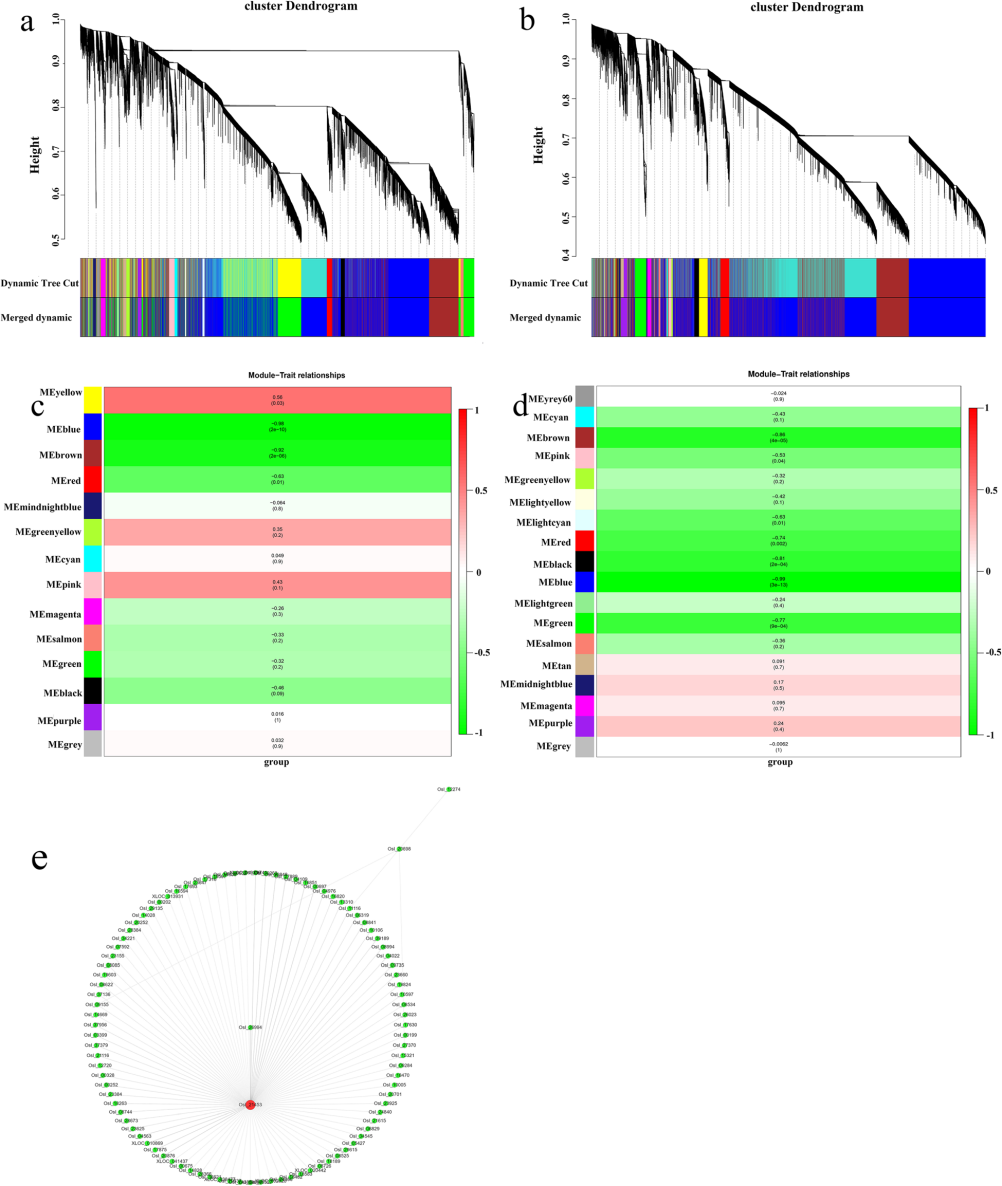
Weighted gene co-expression network analysis (WGCNA) of DEGs identified from 253 and Y12–4 after cold stress. **a** Hierarchical cluster tree showing 18 modules of co-expressed genes in 253. Each of the DEGs is represented by a tree leaf and each of the modules by a major tree branch. The lower panel shows modules in designated colours. **b** Hierarchical cluster tree showing 14 modules of co-expressed genes in Y12–4. Each of the DEGs is represented by a tree leaf and each of the modules by a major tree branch. The lower panel shows modules in designated colours. **c** Module trait relation in Y12–4 and 253. Each line represents a gene module. The number in each Module represents the correlation between modules and traits. The value near to 1, the positive correlation between modules and traits is stronger. The value near to − 1, the negative correlation between modules and traits is stronger. The number in brackets represents the significance *P* value, and the value is smaller, the significance of correlation between traits and modules is stronger. **d** Network of CBF2 and related genes in greenyellow module in Y12–4. Osl_21453 is number of CBF2. Osl_20698 is number of LTG5. Every number is represents one gene

To identify genes related to cold treatment in rice, we performed a weighted gene co-expression network analysis (WGCNA) of all genes. The analysis identified 18 WGCNA modules in 253 and 14 WGGNA modules in Y12–4 (Fig. 6a and b). The *P* value and color gradation of the two modules showed the correlation between the modules. To identify the connectivity of genes related to CT, we performed a heatmap using Student’s t test. The gene eigenvalue of the module showed the comprehensive expression level of module in the samples. A total of 3970 and 5829 genes were found in the “brown” modules of 253 and Y12–4, respectively. Moreover, 1962 genes were found in the two “brown” modules. A total of 218 genes were included in the pathways, including amino acid metabolism, biosynthesis of other secondary metabolites, carbohydrate metabolism, energy metabolism, environmental adaptation, folding, sorting and degradation, translation, signal transduction, membrane transport, nucleotide metabolism, and lipid metabolism. Such genes regulated important pathways for rice adaptation to LT.

Three modules showed a positive correlation (*r* = 0.56, *r* = 0.35 and *r* = 0.43 for yellow, greenyellow, and pink, respectively) with cold stress, indicating that genes in these modules positively regulate cold stress in Y12–4 (Fig. 6c). Seven modules had a negative correlation (*r* = − 0.98, *r* = − 0.92, *r* = − 0.63, *r* = − 0.926, *r* = − 0.33, *r* = − 0.32, and *r* = − 0.46 for blue, brown, red, magenta, salmon, green, and black, respectively) with cold stress, suggesting that the genes in these modules negatively regulate cold stress in Y12–4 (Fig. 6c). In the yellow module, the gene expression levels were upregulated under cold stress in 24 h but downregulated in 0, 3, 6, and 12 h. In the greenyellow and pink modules, the gene expression levels were upregulated under cold stress in 6 and 12 h but downregulated in 0, 3, and 24 h. In the blue module, the gene expression levels were upregulated under cold stress in 0, 3, and 6 h but downregulated in 12 and 24 h. In the brown module, the gene expression levels were upregulated under cold stress in 0 and 3 h but downregulated in 6, 12, and 24 h. In the red module, the gene expression levels were upregulated under cold stress in 0 h but downregulated in 3, 6, 12, and 24 h. In the salmon module, the gene expression levels were upregulated under cold stress in 3 h but downregulated in 0, 6, 12, and 24 h. In the black module, the gene expression levels were upregulated under cold stress in 3, 6, and 12 h but downregulated in 0 and 24 h (Supplementary Figure 3).

Four modules had a positive correlation (*r* = 0.091, *r* = 0.17, *r* = 0.095, and *r* = 0.24 for tan, midnightbule, magenta, and purple, respectively) with cold stress, indicating that tge genes in these modules positively regulate cold stress in 253 (Fig. 6d). Twelve modules had a negative correlation (*r* = − 0.43, *r* = − 0.86, *r* = − 0.53, *r* = − 0.32, *r* = − 0.42, *r* = − 0.63, *r* = − 0.74, *r* = − 0.81, *r* = − 0.99, *r* = − 0.24, *r* = − 0.77, and *r* = − 0.36 for cyan, brown, pink, greenyellow, lightyellow, lightcyan, red, black, blue, lightgreen, green, and salmon, respectively) with cold stress, suggesting that the genes in these modules negatively regulate cold stress in 253 (Fig. 6d). In the lightgreen module, the gene expression levels were upregulated under cold stress in 24 h but downregulated in 0, 3, 6, and 12 h. The 253 genotype showed more negative modules than Y12–4. The positive modules exhibited more positive correlation in Y12–4 than in 253. The result suggests that Y12–4 is a tolerant variety (Supplementary Figure 4).

In the present study, we classified genes *COLD1*, *CTB4a*, *LTG1*, *ctb1*, *ICE1* in the blue module; *qLTG3–1* in the brown module; *CBF1* and *CBF3* in the purple module; and *CBF2* in the greenyellow module in Y12–4 [10, 23–25, 37]. However, *LTT7* was absent in all modules in Y12–4. This results suggests that the genes in the blue, brown, purple, and greenyellow modules are involved in CT. KEGG analysis revealed that the common enriched metabolic pathways are “response to stimulus” and “signaling.” The genes in the blue, brown, purple, and greenyellow modules contributed to CT-related metabolites and plant hormones. The gene response to cold positively correlated with the greenyellow module as detected by WGCNA. CBF2(Osl_21453) is an important transcription factor that regulates the downstream gene response to LT (Fig. 6e).

### Enrichment of GO terms comparing tolerant and sensitive transcriptomes

**Fig. 7:**
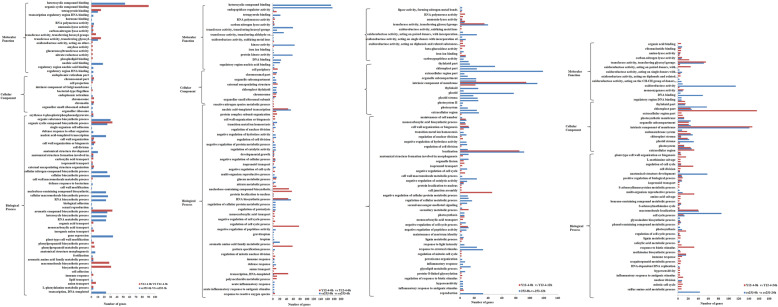
GO slims of functional categorization of the genes during the l response phase. The genes found to be commonly- or genotype-specifically-induced in 253 and Y12–4 during chilling stress of 3, 6, 12, and 24 h. Bars show numbers of 253 (blue) and Y12–4 (red) induced genes

Enriched categories comparing each dataset were searched using GO terms to compare the transcriptomes of germinating seeds from both genotypes (Fig. 7). The GO assignment system was used to obtain functional information for the DEGs, a procedure that can assist in understanding the distribution of gene functions at the macro level. All genes were assigned into three primary GO categories: biological process, cellular component, and molecular function. Thirty-four terms were differentially enriched. Most of the terms enriched in the cold-tolerant dataset in Y12–4-0 h vs. Y12–4-3 h showed upregulated genes, except for rhythmic process, cell junction, and membrane-enclosed lumen. In addition, biological regulation, celluar process, localization, metabolic processs, response stimulus, single-organism process, cell part, extracellular matrix extracellular matrix component membrane, membrane part, organelle, binding, catalytic activity, molecular function regulator, and nucleic acid binding transcription factor activity indicated more upregulated genes than downregulated genes. All terms showed more downregulated genes than upregulated genes, except for structural molecule activity in 253-0 h vs. 253-3 h. Moreover, the model where upregulated genes were greater in number than downregulated genes can be constantly decreased with time when such genes received the same LT stress. The growth, immune system process, metabolic process, molecular function regulator, nucleic acid binding transcription factor activity, and signal transducer activity displayed more upregulated than downregulated genes in Y12–4-0 h vs. Y12–4-6 h. Biological regulation, immune system process, extracellular region part, molecular function regulator, and nucleic acid binding transcription factor activity revealed a uniform trend in 253-0 h vs. 253-6 h. The growth, nucleic acid binding transcription factor activity, signal transducer activity, and structural molecular activity uncovered more upregulated genes in Y12–4-0 h than in Y12–4-12 h. Immune system process and extracellular region part revealed a uniform trend in 253-0 h vs. 253–12 h. Rhythmic process and extracellular region part displayed more upregulated genes in Y12–4-0 h than in Y12–4-24 h. Nucleic acid binding transcription factor activity and extracellular region part revealed more upregulated genes in 253-0 h than in 253-24 h. These terms could be related to known cold-induced processes, such as increased cellulose synthesis, accumulation of unsaturated membrane lipids, osmoregulation, and antioxidant defense activation (Fig. 7).

Annotations were assigned to each transcript and gene by BlastX search against the plant protein collections of four public databases (NR, Swiss-Prot, KEGG, and KOG), with 36 being enriched in the cold-tolerant germination and 53 in the cold-sensitive germination at 0 h vs. 3 h, 61 being enriched in the cold-tolerant germination and 70 in the cold-sensitive germination at 0 h vs. 6 h, 53 being enriched in the cold-tolerant germination and 75 in the cold-sensitive germination at 0 h vs. 12 h, and 81 being enriched in the cold-tolerant germination and 54 in the cold-sensitive germination at 0 h vs. 24 h (Fig. 7).

Interestingly, the terms “carbohydrate metabolic process,” “lipid metabolic process,” “transport,” “ion transport,” “immune response,” “phospholipid binding,” “glucuronosyltransferase activity,” and “antioxidant activity” were found in the cold-tolerant dataset but not in the cold-sensitive dataset at 0 h vs. 3 h. Thus, these mechanisms were potentially involved in the CT observed in the tolerant genotype. Among the terms enriched in the cold-sensitive dataset, “lipid transport” and “defense response to other organism” were observed at 0 h vs. 3 h.

Among the terms enriched in the cold-tolerant dataset, “lipid transport” and “response to oxidative stress” were observed at 0 h vs. 6 h. Among the terms enriched in the cold-sensitive dataset, “defense response” and “response to external stimulus” were regulated many processes at 0 h vs. 6 h.

Among the terms enriched in the cold-tolerant dataset, “response to external stimulus,” “regulation of response to biotic stimulus,” and “oxidoreductase activity” existed at 0 h vs. 12 h. In the cold-sensitive dataset, “defense response,” “response to external stimulus,” and “oxidoreductase activity” were found at 0 h vs. 12 h.

Among the terms enriched in the cold-tolerant dataset, “membrane protein complex” and “response to external biotic stimulus” were observed at 0 h vs. 24 h. Among the terms enriched in the cold-sensitive dataset, “oxidoreductase activity” was observed at 0 h vs. 24 h. The data obtained provided indications of processes that might be involved in the CT mechanism of Y12–4 germination. The cold-tolerant ones triggered other mechanisms that were more efficient in conferring CT. These results might reflect the distinct nature of chilling stress responsiveness in the tolerant and sensitive genotypes. From the 3 h as early-response genes to 12 and 24 h as late-response genes, the genes that regulated the pathway were dynamic and continuous processes. The different GO terms between the tolerant and sensitive genotypes were determined in the 253 and Y12–4.

### Pathway enrichment in different genotypes

The pathway of gene regulation was a dynamic and continuous process. Pathway enrichment is extensively applied to understand the biological progress of rice response to LT. Therefore, we analyzed the pathway enrichment in the terms in 253 and Y12–4. In particular, phemylpropanoid biosynthesis was the most important enrichment among all terms, except in 253-0 h vs. 253-3 h (Supplementary Figure 2). With the extension of cold treatment time, the number of genes of signal transduction pathway increased (Supplementary Figure 2). In addition, a considerable number of genes was found in the metabolic pathways in 253-0 h vs. 253-6 h. However, the number of genes in the single pathway in Y12–4-0 h vs. Y12–4-24 h was greater than that in the three other terms in Y12–4 (Supplementary Figure 2). For genes enriched to plant hormone signal transduction, the important pathway under cold treatment was dropped gradually to top 20 of pathway enrichment in 253. The plant hormone signal transduction pathway was the top 1 in 253-0 h vs. 253-3 h but dropped to top 3, top 6, and top 13 in 253-0 h vs. 253-6 h, 253-0 h vs. 253–12 h, and 253-0 h vs. 253-24 h, respectively. The Q-value dropped as the time decreased. However, the plant hormone signal transduction pathway was the top 2 in Y12–4-0 h vs. Y12–4-3 h but dropped to top 3, top 3, and top 9 in the Y12–4-0 h vs Y12–4-6 h, Y12–4-0 h vs Y12–4-12 h, and Y12–4-0 h vs Y12–4-24 h, respectively (Supplementary Figure 2). The Q-value showed close correlation under cold treatment until 12 h. These results suggested that LT caused dynamic changes in pathway and different regulated genes in the two genotypes. These data suggested that the plant hormone signal transduction was maintained at a high level in Y12–4. Moreover, LT caused the upregulation of brassinosteroid biosynthesis in cold-tolerant germination. The cold-tolerant germination can maintain plant hormone signal transduction under LT more efficiently than the cold-sensitive germination.

### Validation of RNA-seq data by qRT-PCR

**Fig. 8:**
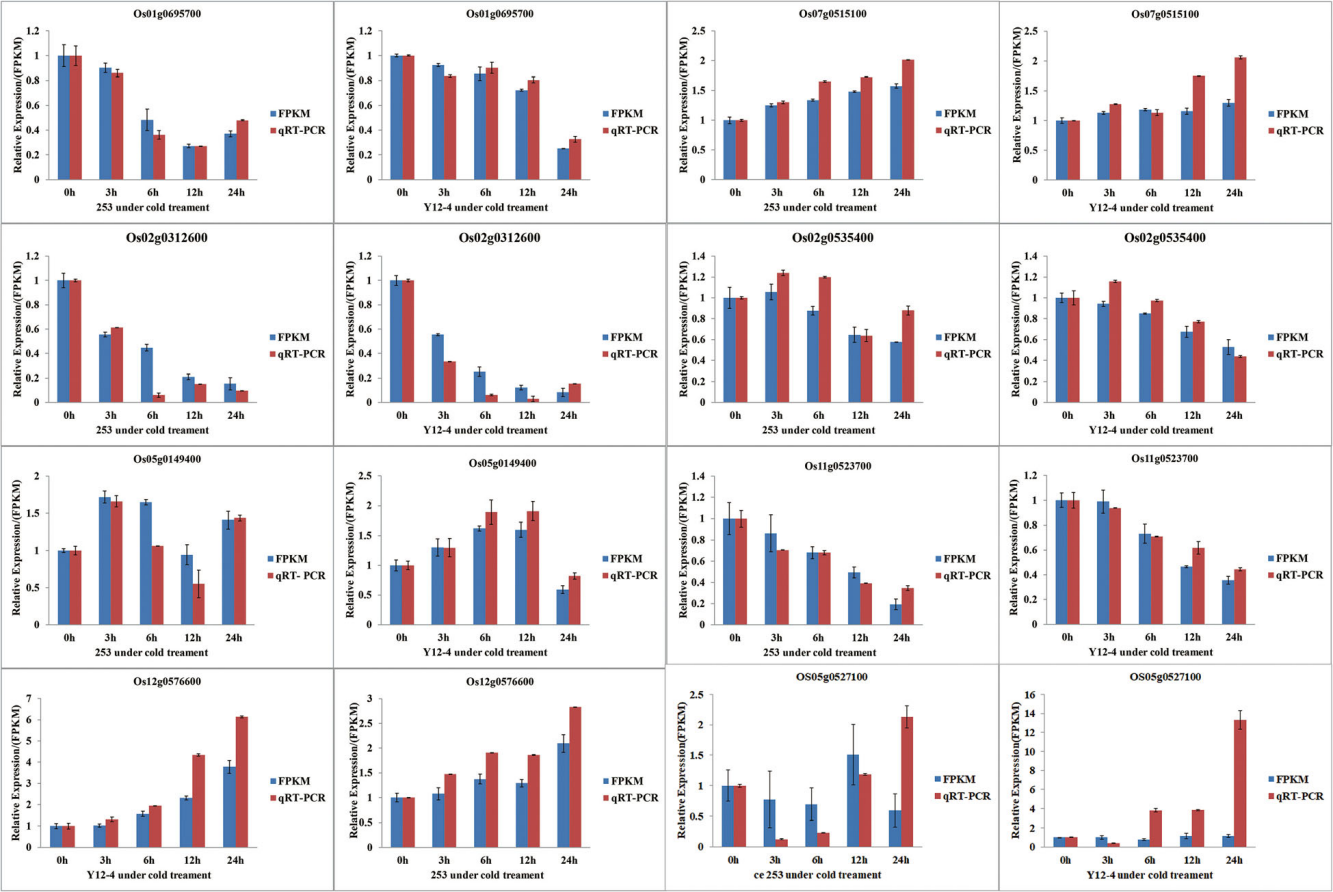
Comparison of genes expression levels using RNA-Seq and qRT-PCR. The relative expression values were normalized to the rice UBQ gene. Error bars indicate standard deviation

Seven genes with remarkably altered expression were analyzed by qRT-PCR to validate the RNA-seq data results. All of these genes were predicted to be related to CT, and functional annotations of these genes are listed in Table S3. The expression patterns of *Os01g0695700*, *Os07g0515100*, *Os05g0149400*, *Os12g0576600*, *Os11g0523700*, *Os02g0312600*, and *Os02g0535400* showed similar expression patterns in the qRT-PCR analysis as in the RNA-Seq analysis. This result indicated the validity of the RNA-seq study (Fig. 8).

### *LTG5* cloning and sequence analysis

**Fig. 9:**
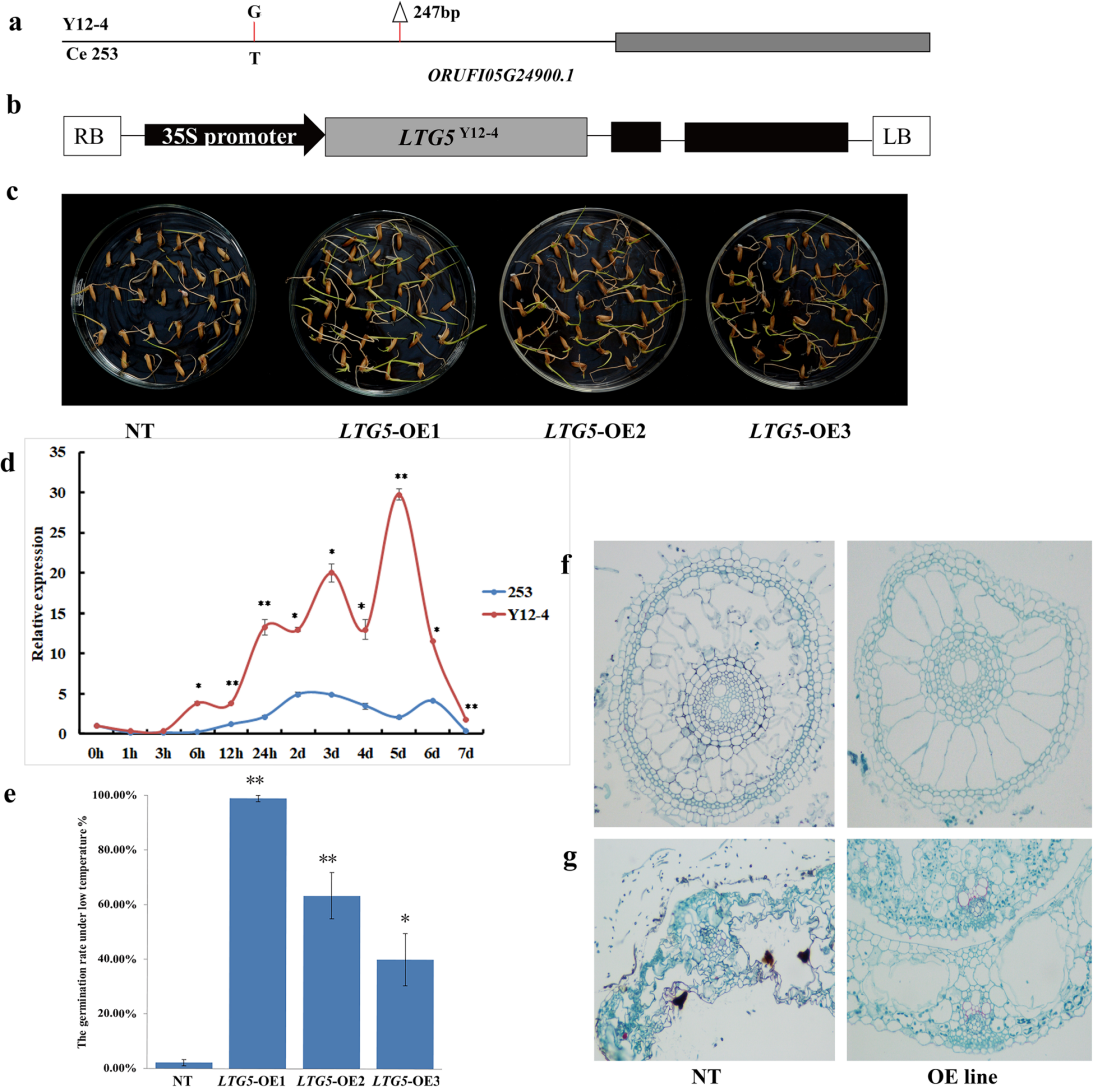
Functional analysis of *LTG5* at the germination stage. **a** Phenotype of plants and panicles of overexpression lines grown under cold stress. **b** Statistical results for seed setting of NT and *LTG5*-OE line under cold stress. Data represent means±s.d. (*n* = 15). **c** Observation on the microstructure of root of NT and *LTG5*-OE line. **d** Observation on the microstructure of leave of NT and *LTG5*-OE line

Basing from the nucleotide polymorphisms identified by RNA-Seq, we selected three genes from the WGCNA brown and greenyellow modules with high relative expression and different expression patterns between 253 and Y12–4 for cloning and functional indetification. The results of WGCNA imply that *CBF2* regulates *LTG5* in the greenyellow module (Supplementary Figure 5). *LTG5* was responsible for encoding UDP-glucosyltransferase and for the sensitivity to LT from the greenyellow module in Y12–4. One pair of primers (Appendix S1 in Supplementary materials) was designed to amplify *LTG5* ORFs from “Y12–4” (Appendix S2) of *O. rufipogon Griff*. on the basis of other available Nipponbare *LTG5* nucleotide sequences (Genbank accession: *ORUFI05G24900.1*). One SNP and one Indel were found in the promoter region of *ORUFI05G24900.1* between Y12–4 and 253. The expression of *LTG5* in Y12–4 played an important role on CT at the germination stage (Fig. 9).

### *LTG5* regulates CT at the germination stage

Several experiments were conducted to estimate the the function of *LTG5*. First, an overexpression line containing the *LTG5* from Y12–4 driven by the 35S promoter from tobacco cauliflower mosaic virus (CaMV35S) was separately introduced into 253. Reverse-transcription PCR indicated that independent T0 transgenic lines showed different expression levels of *LTG5*, and the gene expression in T3 lines was also verified by qRT-PCR. Three overexpression transgenic lines of each *LTG5* were used for further analyses. OE-*LTG5* showed stable and evidently enhanced CT compared with non-transgenic lines (NT) under LT conditions. Compared with the NT, the germination rates of OE-*LTG5*–1, OE-*LTG5*–2, and OE-*LTG5*–3 under LT enhanced by 96.97, 61.11, and 37.78%, respectively (Fig. 9). In the leaf of OE-line, bulliform and parenchymal cells were broken, but mesophyll tissue, vascular bundle, and mechanical tissue retained their structure and morphology under cold stress. However, bulliform cell, mechanical tissue, and parenchymal cell were broken in the leaf of NT (Fig. 9).

### *LTG5* expression pattern and subcellular localization of its gene product

#### The expression of LTG5 was detected in various tissues by fluorescence quantification to detect the temporal and spatial expression patterns of LTG5

**Fig. 10:**
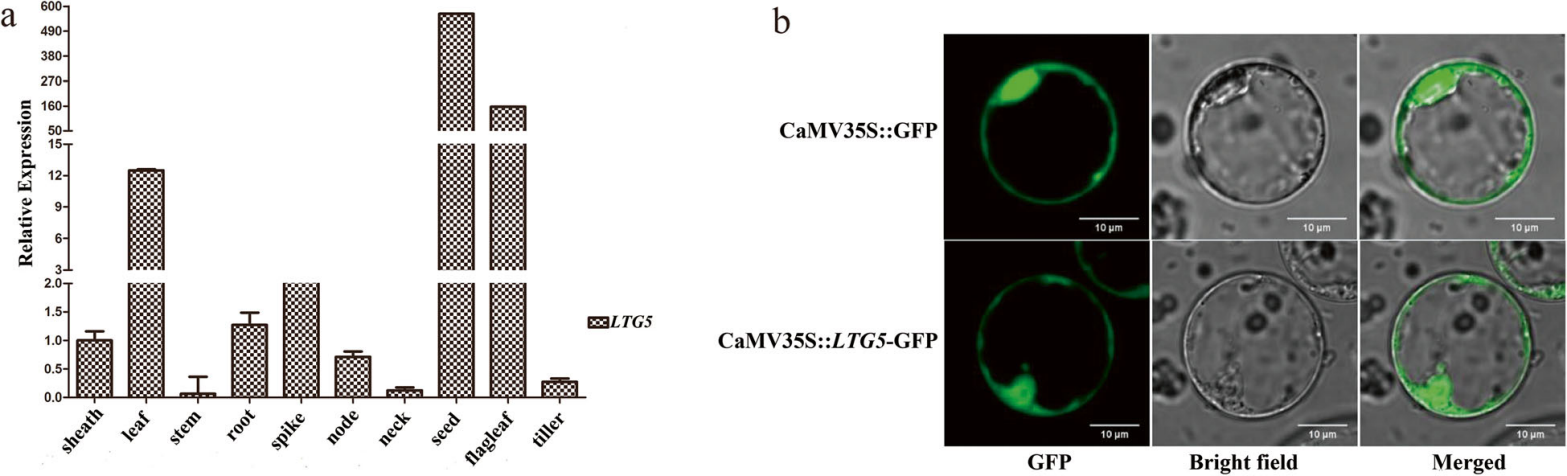
Expression in the tissue and Subcellular localization of *LTG5*. **a** Expression in the tissue of *LTG5*. **b** Subcellular localization of *LTG5*

The expression of *LTG5* was detected in many rice tissues used for qRT-PCR to confirm the spatial expression patterns of *LTG5* (Fig. 10). *LTG5* was widely expressed in several rice tissues, such as the sheath, leaf, stem, root, spike, node, neck, seed, flag leaf, and tiller tissues. The highest expression of *LTG5* was detected in the seeds (Fig. 10). *LTG5*-green fluorescent protein (GFP), a fusion protein, was expressed under the control of the 35S promoter in rice leaf protoplasts to ensure the subcellular localization of *LTG5*. As shown in Fig. 10b, the GFP fluorescence in 35S:*LTG5*-GFP transgenic protoplast cells was observed exclusively in the membrane. As such, this result suggests that *LTG5* is a membrane and nucleus-localized protein, consistent with its proposed function in regulating growth and development in rice.

## Disccussion

Rice originated from tropical or subtropical areas. Cold stress is a major abiotic stress limiting rice productivity. Therefore, studying the mechanisms underlying the cold stress response of rice is important. Wild rice in Guilin of Guangxi possesses strong CT at the germination stage and is an excellent resource for molecular breeding of cold-tolerant rice [38–40]. In the current research, RNA-Seq analysis was used to evaluate the transcriptomes of the cold-tolerant and cold-sensitive rice genotypes at the germination stage following cold treatment. The cold-tolerant wild rice Y12–4 showed fewer DEGs than the cold-sensitive rice 253. The number of DEGs increased in the two rice genotypes under LT. Plant hormone signal transduction was maintained at a high level in Y12–4. A novel gene, *LTG5*, which encodes an UDP-glucosyltransferase, was identified. The gene improved the CT in rice at the germination stage.

### RNA-Seq analysis of the dynamic response mechanism in rice under cold stress

RNA-Seq is a powerful tool for identifying the transcriptomic changes in the response mechanisms of rice to cold stress. The present study provided a comprehensive transcriptome survey to understand the genes/processes responsive to LT at the germination stage in rice. Cold responses are complex traits that involve many signal pathways, transcriptional regulators, and metabolites [41].

More DEGs downregulated the response mechanisms in cold-sensitive rice 253 than in cold-tolerant rice Y12–4 under cold stress. The number of DEGs increased with the time of cold stress exposure. The modules had more positive correlation in Y12–4 than in 253. Maia et al. showed more alignment genes in sensitive genotypes and revealed 19 times more DEGs in the cold-sensitive genotype than in the cold-tolerant genotype in response to cold. In addition, the signal transduction-related genes of calcium-binding EF-hand, mitogen, activated protein kinases, cyclin-dependent kinase, C2H2-type zinc finger, DREB, and WRKY are upregulated in Oro but downregulated in Tio Taka [9]. Shen et al. showed that the DEGs of regulation of nucleobase-containing compound metabolic process, regulation of nitrogen compound metabolic process, and regulation of primary metabolic process are upregulated in three cold-tolerant genotypes, whereas the DEGs of serine family amino acid metabolic process, M phase, and histone modification are down regulated in the cold-tolerant genotypes [17]. In the present study, the DEGs of membrane part, intrinsic component of membrane, and localization were upregulated in Y12–4.

The upregulated genes in Y12–4 included calmodulin family protein, cytochrome P450 family protein, ethylene response factor, jasmonate zim-domain protein, NB-ARC domain containing protein, serine/threonine protein kinase, cold shock protein, MYB transcription factor, UDP-glucosyl transferase family protein, and WRKY gene. More genes were involved in signal transduction and energy metabolism. *COLD1* [23], *CTB4a* [10], *LTG1* [25], *ctb1* [37], *ICE1* [42], *qLTG3–1* [24], and *CBF2* [32] can be detected in the positive and negative correlation modules. Evidently, many genes responded to cold stress rapidly and positively in the cold-tolerant genotype in a short exposure time. Calcium signaling cascades in the sieve element cytoskeleton and MAPK cascade pathways [43]. Ca^2+^ can induce the expression of *CBF*/*COR* genes and transcription factors (MYB) in the cold-signaling pathway [42, 44].

However, downregulated genes in the cold-tolerant genotype, which included cupredoxin domain-containing protein, glycoside hydrolase, and Harpin-induced 1 domain-containing protein, were similar to peroxidase. ROS generatuon is triggered by biotic and abiotic stresses. Oxidative stress destroys cell membrane and limits rice vegetative growth [45].

In the cold-tolerant genotype exposed to cold stress for 24 h, the upregulated genes contained abscisic acid (ABA) stress-ripening inducible protein, calmodulin family protein, jasmonate zim-domain protein, serine/threonine protein kinase, UDP-glucuronosyl/UDP-glucosyltransferase family protein, and so on. The ABA signaling pathway was involved in the regulation of cold stress response that was transduced by ABF1/2 and the ABRE-containing OsNAC gene in rice. *OsPYL10* overexpression regulates its ligand ABA accumulation to improve the drought and cold stress tolerance of indica rice [46]. *CTB4a*, which encodes a serine/threonine protein kinase, improves rice CT at the booting stage [10]. JAs shows a complex relationship between the CBF pathway and counteracted chilling stress by inducing ROS avoidance enzymes [47].

Gene expression is a dynamic and continuous process by inducing cold. Cold stress can influence cell membrane fluidity and cytoskeleton depolymerization [29]. Calcium signaling activates CaMs, CMLs, CBLs, and CDPKs protein with the EF-hand domain, which regulates downstream signaling [48–50]. Genes of signal transduction showed more vitality at an early stage, and long-term cold stress induced functional gene upregulation. Different types and numbers of genes were induced by long-term cold stress. The resistance mechanism to LT was upregulated. Moreover, LT destroyed the whole system in rice. The current research offered insights into the dynamic response mechanism to LT between the cold-tolerant wild rice and cold-sensitive indica and into the particular genotype that responds to cold stress.

### *LTG5* role at the germination stage under cold stress

Plants need UDP-glucosyltransferase to glycosylate plant hormones and all major classes of secondary plant metabolites [51]. Glycosyltransferases can catalyze the addition of sugar to bind to different receptor molecules, such as proteins, nucleic acids, oligosaccharides, and lipids. Glycosylation of aglycones can alter the activity, solubility, and transport of glycosyltransferases [52]. In *Arabidopsis thaliana*, the hydrogen peroxide-responsive UDP-glucosyltransferase UGT74E2 is involved in the modulation of plant architecture and water stress response [53]. The potato glucosyltransferase gene promoter increases enzyme activity by LT, which indicates that the promoter plays a regulatory role in plant protection against abiotic stresses [54]. The Arabidopsis glucosyltransferase UGT71B6 influences ABA and the related ABA metabolites and ABA glucose ester (ABA-GE) [30]. In potato, the UDP-glucosyltransferase gene is highly correlated with glucose and gene expression under cold-induced sweetening [55]. Genome-wide identification of WRKY genes and their response to cold stress in *Coffea canephora* showed that UDP-glucosyltransferase activity is remarkably represented [56]. The overexpression of *LTG5* increases the CT of rice at the germination stage. *LTG5* is a conserved UDP-glucosyltransferase in plants. The present results suggest that *LTG5* has a great potential for improving rice CT at the germination stage via molecular breeding techniques.

## Conclusion

We provided an overview of different molecular changes between cold-tolerant common wild rice and cold-sensitive indica under LT stress. RNA-seq data indicated that the transcription in response to cold relatively differed between the tolerant and sensitive genotypes. Our transcriptome data can be used to identify novel targets for CT. In addition, the present study discovered the cold-tolerant gene *LTG5* from wild rice. *LTG5* encoded a UDP-glucosyltransferase and played an important role in regulating the germination rate under cold stress. However, further studies were needed to establish this connection between gene expression and cold response. Therefore, a unique level of mRNAs under cold treatment and *LTG5* may be valuable for in-depth analyses and further applications to understand novel regulatory mechanisms for CT in rice-germinating seeds.

## Methods

### Plant materials and cold treatment

The cold-sensitive indica rice variety ce253(253), which is widely planted in Guilin, Guangxi Province, China, and the cold-tolerant common wild rice (*O. rufipogon Griff*.) Y12–4 were used in the present study. All plant materials were preserved in Rice Research Institute, Guangxi Academy of Agricultural Sciences, and Y12–4 was obtained from national germplasm Nanning Wild Rice Nursery. Seeds were washed three times with distilled water and then soaked in 70% ethanol for 5 min. Then, the seeds were soaked in 5% sodium hypochlorite for 15 min and washed three times with distilled water before germinating in a glass dish (9 cm).

All rice seeds were germinated in a greenhouse at a temperature of 28 °C ± 2 °C, relative humidity of 80–100%, and photoperiod of 12 h. The seeds were prepared with a coleoptile length of ≥5 mm. The three day-old seeds sampled at 0, 3, 6, 12, and 24 h under 4 °C and their corresponding rice sampled at 0, 3, 6, 12, and 24 h were subjected to total RNA extraction and RNA-Seq analysis. Three biological replicates (each one containing at least 30 germinating seeds) were collected, immediately frozen in liquid nitrogen, and then stored at − 80 °C until further analysis.

One method was adopted to evaluate CT at the germination stage. Three-day-old seeds were treated under LT (4 °C) for 10 days and recovered for 5 days. Phenotypes were evaluated by survival rates. At least five pots of 30 plants were assessed for each line. Germination tests were conducted as described by Fujino et al. [57] with minor changes.

### RNA-Seq and data analysis

The total RNA of each sample was extracted using TRIzol R reagent (Invitrogen, Carlsbad, USA) following the manufacturer’s instructions. RNA was treated using PrimeScript™ RT reagent Kit with gDNA Eraser for further analysis. Quality control was confirmed within the Illumina HiSeq software. Reads were mapped to the reference genome sequence of Nipponbare. All reads of gene passing the filtering specifications were mapped onto the reference genome by HISAT2, Tophat2(2.1.1) and IRGSP-1.0. Genes are available at the Rice Genome Annotation Project (http://rice.plantbiology.msu.edu) with a perfect match or one mismatch.

Genes with a false discovery rate below 0.05 and fold change≥1.5 were considered differentially expressed genes/transcripts. The DEG loci in the cold-tolerant or cold-sensitive datasets were performed to find enriched GO enrichment, and the groups were considered to be significantly enriched. Known and newly predicted genes were annotated based on three public protein databases to obtain comprehensive information on the detected transcripts. DEGs were identified in each pair of samples using the criteria listed in the Materials and Methods section (Fig. 3).

Clustering of genes with similar expression patterns is an analytical strategy that helps identify the function of unknown genes or characterize the unknown functions of known genes. Gene expression levels were normalized to fragments per kb of transcript sequence per million base pairs sequence (FPKM) to identify the DEGs in the two rice genotypes [11]. The fold change of DEGs was calculated using the FPKM value of the treatment vs. the control. The cDNA libraries were sequenced on the Illumina sequencing platform by Genedenovo Biotechnology Co., Ltd. (Guangzhou, China).

### Vector construction and genetic transformation

For the construction of an overexpressed plasmid, the full 1.4 kb exons and intron region of *LTG5* were amplified from Y12–4, digested with PacI and KpnI, and cloned into binary vector pMDC32 [58]. All fragments were amplified by the high-fidelity PCR enzyme KOD-FX (TOYOBO, KFX-101). Primer sequences for vector constructions were provided in Appendix S1. All plasmids confirmed by sequence were introduced into *Agrobacterium tumefaciens* strain EHA105 and transferred into recipient materials by using the Agrobacterium-mediated method [59].

### qRT-PCR analysis of the candidate genes

The cold-sensitive line 253 and cold-tolerant wild rice Y12–4 were used to identify the expression patterns of the putative candidate genes. The RNAs of rice for qRT-PCR were extracted using TRIzol R reagent (Invitrogen, Carlsbad, the USA) following the manufacturer’s instructions, and PrimeScript™ RT reagent Kit with gDNA Eraser was used in all RNAs for further analysis. Seeds germinated with normal temperature served as the control. The sprouts of the two lines were collected at 0, 3, 6, 12, and 14 h under LT and normal temperature for three repetitions. The samples were placed in liquid nitrogen immediately and then stored at − 80 °C for total RNA isolation. We constructed a single-end cDNA library for each rice genotype. The first-strand cDNA was synthesized using the PrimeScript™ RT reagent Kit with gDNA Eraser (Perfect Real Time). As an endogenous control, the housekeeping gene UBQ (*Os03g0234200*) was used as the reference gene, and the ΔΔCt method was used to measure the relative expression values [60]. Primer Premier 6 software Primers for each gene were designed using Primer Premier 6 (Appendix S1). qRT-PCR was performed with a BioRad CFX96™ Real-time System C1000 using SYBR® Premix Ex TaqTM (Tli RNaseH Plus, TaKaRa, Japan) in accordance with the manufacturer’s protocol.

### Subcellular localization

The plasmids CaMV35S::GFP and CaMV35S::*LTG5*-GFP were transformed into rice leaf protoplasts for subcellular location. GFP was excited with a 488 nm laser.

## Supplementary information

**Additional file 1: Supplementary Figure1.** Correlation analysis among the sample treatments

**Additional file 2: Supplementary Figure 2.** Pathway enrichment in different genotypes

**Additional file 3: Supplementary Figure 3.** Module Gene expression pattern of module in Y12–4, red means upregulated genes, green means downregulated genes

**Additional file 4: Supplementary Figure 4.** Module Gene expression pattern of module in 253, red means upregulated genes, green means downregulated genes

**Additional file 5: Supplementary Figure 5.** qRT-PCR of candidate genes and germination rate under low temperature of OE-line for candidate genes

**Additional file 6: Table S1.** genes were significantly differentially expressed in 253.

**Additional file 7: Table S2.** genes were significantly differentially expressed in Y12–4.

**Additional file 8: Table S3.** Genes for qRT-PCR.

**Additional file 9: Appendix S1.** primers in this study.

**Additional file 10: Appendix S2.** Seqence of *LTG5* from “Y12–4”.

## Data Availability

Relevant data are within the paper and its Supporting Information files, and sequence data is deposited in the Nation Center for Biotechnology Information (SRA) database with accession numbers SRR11248805, SRR11248806, SRR11248807, SRR11248808, SRR11248794, SRR11248800, SRR11248801, SRR11248802, SRR11248803, SRR11248804, SRR11248797, SRR11248796, SRR11248795, SRR11248822, SRR11248798, SRR11248799, SRR11248793, SRR11248809, SRR11248810, SRR11248811, SRR11248812, SRR11248813, SRR11248814, SRR11248815, SRR11248816, SRR11248817, SRR11248821, SRR11248818, SRR11248819, SRR11248820 (for a total of 30 sequences). All other data generated or analyzed during this study are included in this manuscript.

## Abbreviations

DEGs: Differentially expressed genes
RNA-seq: RNA Sequencing
qRT-PCR: Quantitative real time polymerase chain reaction
*LTG5*: Low temperature growth 5
LT: Low temperatures
ROS: Reactive oxygen species
CBF: C-repeat binding factor
GO: Gene ontology
CT: Cold tolerance
*COLD1*: Chilling tolerance divergence1
*CTB4a*: Cold tolerance at the booting stage
MAPK: Mitogen-activated protein kinase
OsCPK24: Ca^2+^-dependent kinase24
253: Ce253
FDR: False discovery rate
FPKM: Fragments per kb of transcript sequence per million base pairs sequence
WGCNA: Weighted gene co-expression network analysis
CaMV35S: Cobacco cauliflower mosaic virus
OE: Overexpression
NT: Non-transgenic lines
GFP: Green fluorescent protein
ABA: Abscisic acid
