# Supplementary figures and images for: Transcriptomic profiling of germinating seeds under cold stress and characterization of the cold-tolerant gene LTG5 in rice
Source: BMC Plant Biol. 2020 Aug 6;20:371. doi: 10.1186/s12870-020-02569-z (PMC7409433; doi:10.1186/s12870-020-02569-z)

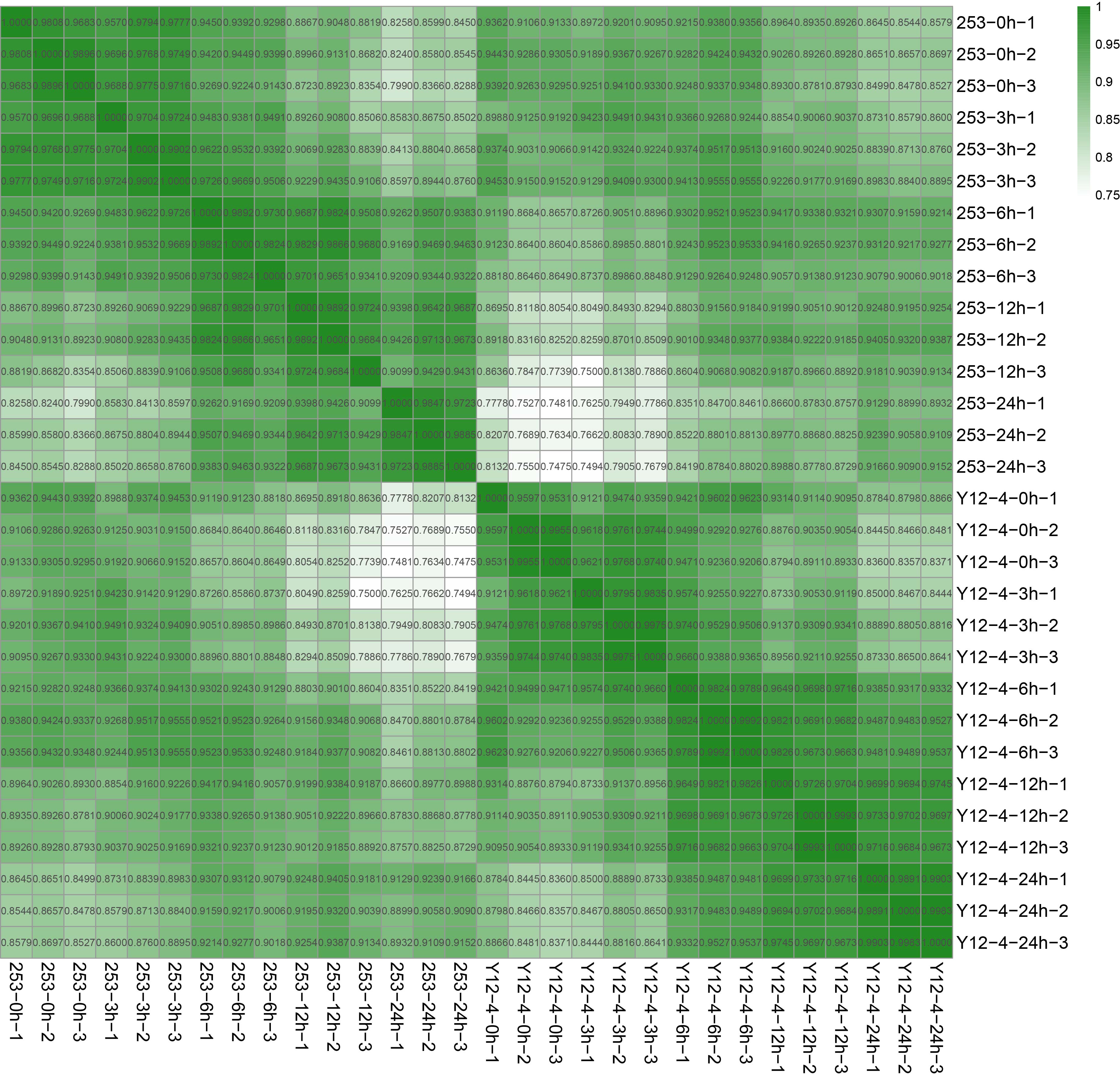

Supplement: Supplementary file 1 — Additional file 1: Supplementary Figure1. Correlation analysis among the sample treatments [file 12870_2020_2569_MOESM1_ESM.jpg]

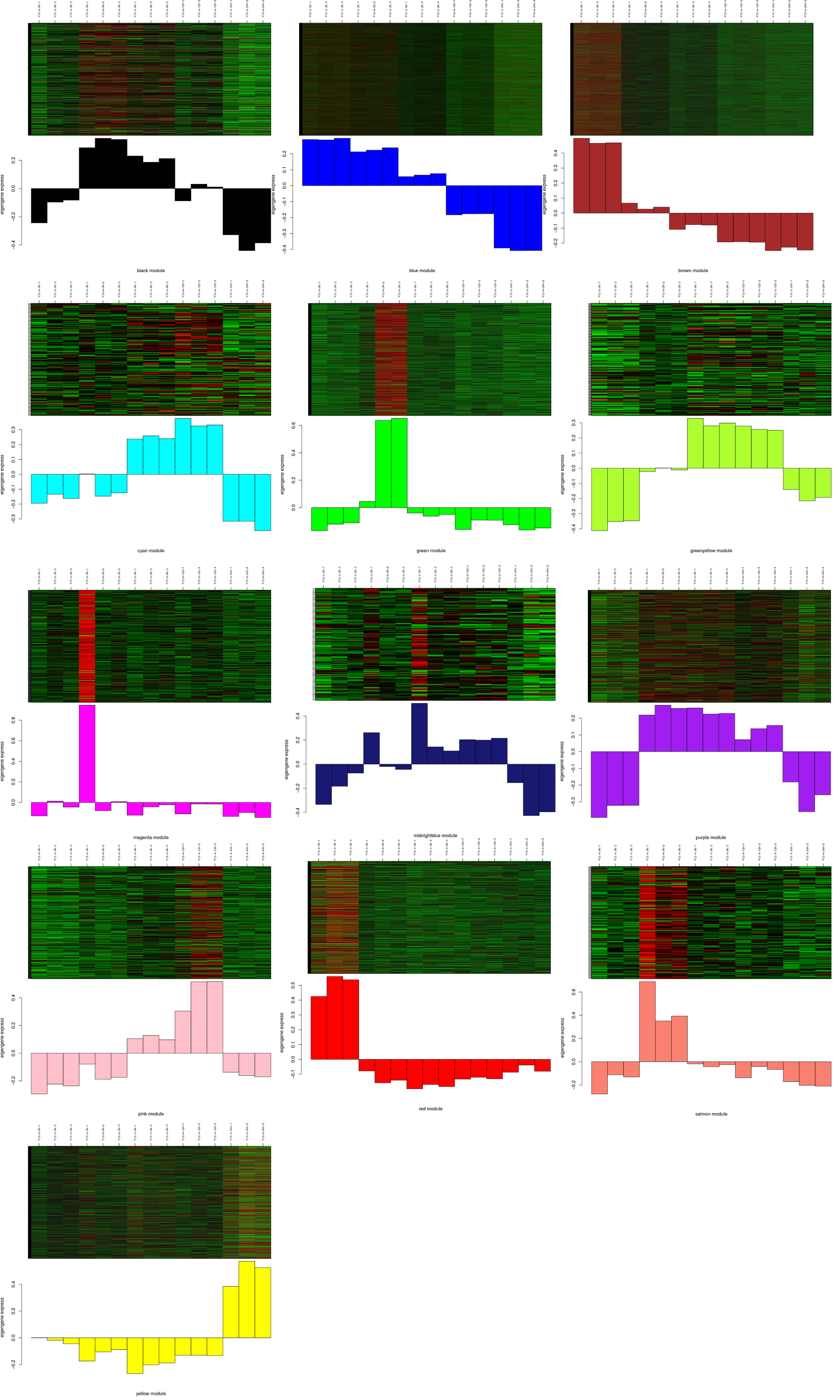

Supplement: Supplementary file 3 — Additional file 3: Supplementary Figure 3. Module Gene expression pattern of module in Y12–4, red means upregulated genes, green means downregulated genes [file 12870_2020_2569_MOESM3_ESM.jpg]

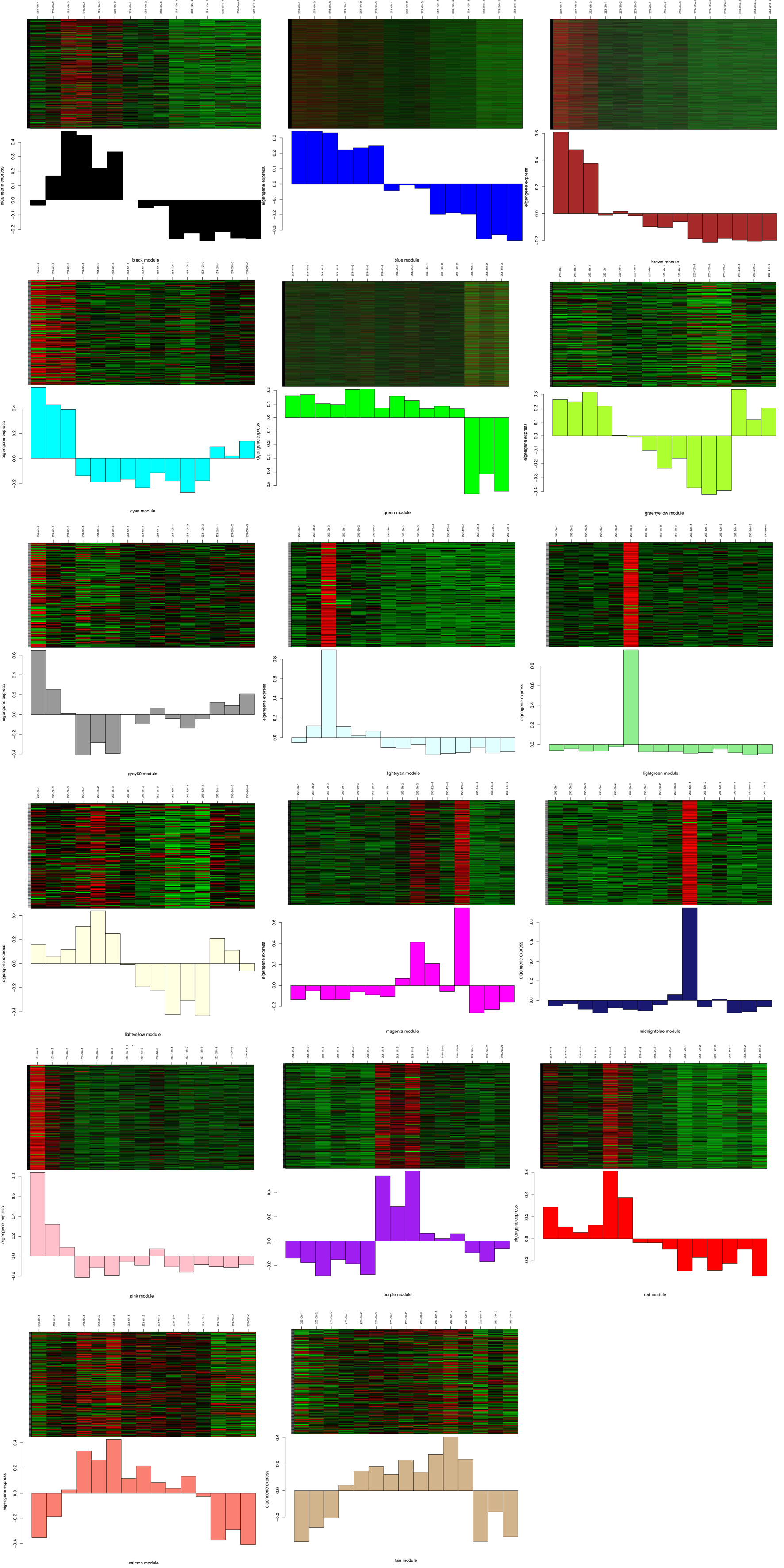

Supplement: Supplementary file 4 — Additional file 4: Supplementary Figure 4. Module Gene expression pattern of module in 253, red means upregulated genes, green means downregulated genes [file 12870_2020_2569_MOESM4_ESM.jpg]

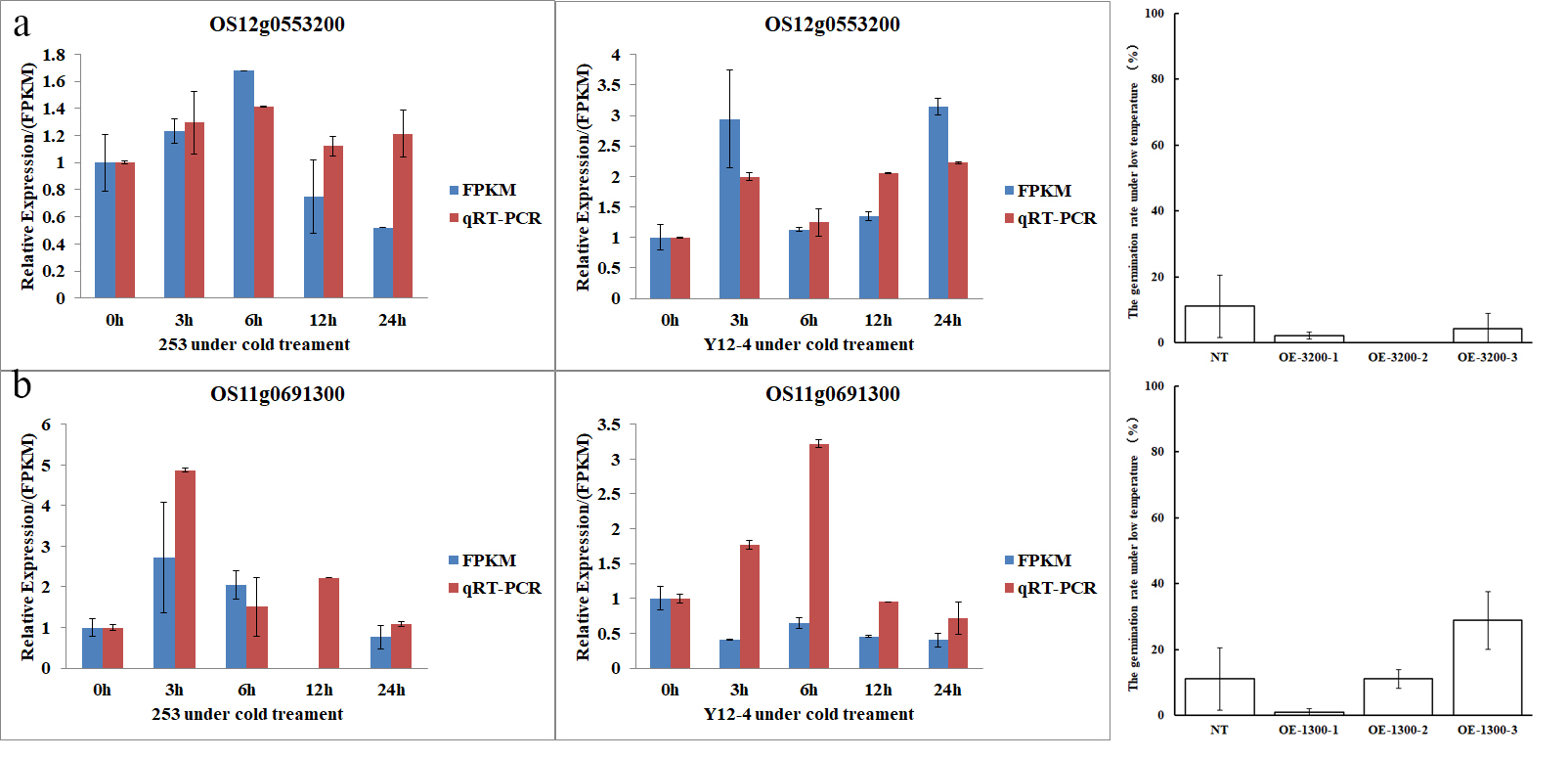

Supplement: Supplementary file 5 — Additional file 5: Supplementary Figure 5. qRT-PCR of candidate genes and germination rate under low temperature of OE-line for candidate genes [file 12870_2020_2569_MOESM5_ESM.jpg]
