# Supplementary material for: Transcriptomic profiling of germinating seeds under cold stress and characterization of the cold-tolerant gene LTG5 in rice
Source: BMC Plant Biol. 2020 Aug 6;20:371. doi: 10.1186/s12870-020-02569-z (PMC7409433; doi:10.1186/s12870-020-02569-z)
Supplement: Supplementary file 10 — Additional file 10: Appendix S2. Seqence of LTG5 from “Y12–4”. [file 12870_2020_2569_MOESM10_ESM.docx]

**Sequence of *LTG5* From y12-4**

GCAGGTTTAAACGAATTGGCCCTTCTCCTCCTTTCAACCAATCACAATCATTCTCCTTCACCTATTTTTTCTTTTCAACCAATCACACACCTTCTCTAATCATTCTCACCTACTTTCTTAATACCAGTGCCAACCTTGAAAATACCTACAATTTAAAACGGAGGAATTATTTATCACCCTATTCATCTACATGTGCCCTCCGTCCCAGAATAACCGTTTCAGGAATTGTCACTTCAGTTCAGATTTTTTGTCCCTAACCCAGCAGGCCAGTTAGTAGTCACACGTGAACGTTCCTGCTCGCTGTTCGTAATACGGTAGATGTG.TTTTTTTTTTTAATCTCCAGGATCAGTAATGATACCACCAGTCTCAGAAGATATATAGTATATGTAGATGAGTTAAATTTCCACCCCCCCTCCGCAAAAAAAAAGTTAAATTTGTTATTTTATATTTATTACTCATATATTAAATTTGAATTTAAATGTTTATGGAATGAAATATATTATATTGATATATCATGTCAGTTTTTTAATATATATAATAACTATTCGAATACTACCTCCGTCTCAAAAAGTAGTTTTGCACTACTTATATACAACGTTTGACCGTCCGTTTTATTTG.AAAAAAAATTATGATTCGTATTTTTGTTGTTATTAGATAATAAAACATGCATAGTACTTTATGTGTAATTAATTTCTTTTAAATTTTTTATATTTTTTTAGATAAGACGGGCGGTCAAATGTTGAACACGGAAATCCACGACTACAGTTAATATGGGACGGAGGTAGTACCATATACTCCCTCCGTCAAAAAAAAATAATCTAGTATTAGTATTAGATGTGATATTTTTAAGTACAACGAATTTGGACAAAAGTCTATCTAGATTCATTGTTGTATTAGAAAATGTCCAATCCAACTAATTTAACTTATACAAGTTGAAACAAACATAACATATTTAACTTTGTATTTAATGTGAATTTTCACATTCAAAATTATTATTTTGTTTATACAATTATTTTGGTTTCTAATTGTATAGATGGAATTTGAACTTGCTCGTGTATAGTCAGTCATATATCACATATTGATCTATCTTATTAATTTTTTTTAAAAAAATAGTGACTTTTTAATCCACATGCACAAAACGAGTAATGTCTCATAGGAAAAAAAATCCAGCACCATCTACACGTAACCCTCAATCCTCCCAATATAATATAACGAAGCCAATCCCAACAAGGGCGACATGGGGCATGTAACGGCCGGGCTACAAACCGTGTAAAAAATTGAAGGGATTATCTTAAAAAAACTGCTGCAAGTTAATGGCTCTGAATAGTTTTTTTTTTTGGTCTTTTTTGTTTTCGTTGGATATACTTCCGTATCGGTTTCCAAAAATTGTCTTAATTATTCCCTCCATATTTATTTTGAGATAGAGGTTGGTAGCTCGGTTTGGATCCGTTTTGTAGCAAGTTACCCGGCCGATATTCAACTGATCGCTGTTTGTAGTAATACGGGAAGATGTGATTTTCTTTTTAATCTCCAGGATAAAAATGATACAGCGGTCGAGCTTTGGTTGGAGGAAGGACTATAGCCTATAGGGAAAACGCCGTTTGCCTTTGTCCTTTGAATGCTTTTGTTGTGAAGCTGGCAGCGGTCGGTCACCTTCGATCGGATGCATTTCTGATTCTTTCTTCTTGGAAAAGAAGGGCTAAAGATTGTCCAACGGGGCACGGTGCTGTAGTATGGGACAACGGCAGATGATGTGAACCCGTATGACTGTATTTCTAATTCTGTGTCACGATCACCGGACTAATTGACTTTCCCGACTAATAATAAACTCTATCACGATCTCTATCTGATTCGTCGTGTCCTTATTTTACTCCTGTGGACATCACCACCGACGGCGGTTCCT..AAAACAATCACGCTTGTGAATCTCGAGGCCTACCGGCCAATTAATTTAATCACATCTGTTACTCCCTCAAGTAAAAAAAGACAAACCCTAAATTCCATGTCCAACGTTTAACCGTCCGTTTTATTTAAAAAATTAAAAAGATAAGTCACGCATAAAATATTAATCATGTTTTATCATCTAACAACAATAAAAATACGAATTATAAAAATTTTCATATAAGACGGAGAGTCAAACGGAAACTCAGAATTTATTTTTTTTTCAAACAGAGGAAGTAGTTGGTGGCGGCACAGACGAACGACTAGAAGCCATCGATGTGTATCCTGCCTTGCTTGCGTGGCTGCGTGTGATGATCATACTGTGATCCCATCTTGTGCAGTACTAGTATACGCCAGACGTGAGCTCCCTTCCTTCCTCGTCTACCAGTTTACTGAACTGCTGACTTCAGTGAAAGAGTAGTCCTCCTAATTCCTCCAAAACTTTATAAAGCCAACGTGAAATTTGTATTGGTTGTGCAGGTCACATTGGGGCGTGTCAACGTTGCTCACCATGGCTTACCATTCAACCCCTCTATATATACTTGTGGTTGTCCGATTTGAGGGCATTTGTTTCCGTCTTTTTTACATCCCGTTTGACCGGAACAGCAGCTAGCCATGACGACAAACACGTTTGTGCTGTTCCCGTCGCTGGGCGTCGGCCACCTGAACCCCATGGTGGAGCTAGCCAAGCACCTGCGCCGCCGCGGCCTCGGCGTCATCATCGCGGTGATCGATCCGCCCAACAACGACGCCATGTCGGCCGACGCGATGGCGCGCCTCGCCGCGGCCAACCCTTCCGTCACGTTCCGCATCCTGCCGGCGCCGGCCAGCCCGGACCCCGGCGCGCACCATGTCAAGCGCAACCTCGACACGCTCCGGCTCGCCAACCCCGTGCTCCGCGAGTTCCTCCGCTCCCTGCCCGCCGTCGACGCACTCCTGCTCGACATGTTCTGCGTCGACGCGCTCGACGTCGCGGCCGAGCTCGCCATCCCCGCCTACTTCTTCTTTCCCTCCCCGGCCAGCGTCCTCGCCGTCTTTTCCCACCTCCCGTATTACTACCGCAACGCGCCGTCGTTGAGGGAGATGGATAAGGCGGCGCTCATCCGATTTCCCGGCATTCCGCCGATCCGCAACGTCGACATGCTGGCCACGGTGAAGGACAAGGAGAGCGAGACGACCAAGATCAGGTTGTACCAGTTCAAGCGGATGATGGAAGGGAAGGGCGTGCTGGTGAATAGCTTCGACTGGCTGGAGCCCAAGGCCCTGAAAGCGCTCGCCGCCGGTGTCTGCGTGCCCGACATGCCCAAGCCCAGAGTCTACTTAATCGGGCCACTGGTCGACGCCGGCAAGAAGATAGGGAGCGGCGCCGAGAGGCACGCGTGCCTCCCGTGGCTTGACGCCCAGCCGCGGCGGAGCGTCGTGTTCCTCTGCTTCGGCAGCCAGGGCGCGTTCCCGGCGGCGCAGCTGAAGGAGTTAGCTCATGGGCTGGAGAGCTCCGGCCACCGATTCCTGTGGACCGTGAGGAGCCCACCGGAGGAGCAGTCCACATCACCGGAGCCGGACCTGGAGCGGCTGCTTCCGGCGGGGTTCTTGGAGAGGACGAAGGGCAGAGGCATGGTGGTCAAGAACTGGGTGCCACAGGCGGAGGTGGTGCAGCACGAGGCGGTAGGCGCGTTCGTGACGCACTGCGGGTGGAACTCGACGCTGGAGGCGATCATGTCGGCGCTGCCGATGATATGCTGGCCGCTGTACGCGGAGCAGGCGATGAACAAGGTGATCATGGTGGAGGAGATGAAGATCGCCGTGCCGCTTGACGGGTACGAGGAAGGAGGGTTGGTGAAGGCCGAGGAAGTGGAGGCGAAGGTGAGGCTGGTGATGGAGACCGAGGAAGGGAGGAAACTCAGGGAGAAACTGGTGGAGACGAGGGACATGGCGTTGGATGCCGTCAATAAGGGTGGGTCTTCTGAAGTGGCATTTGATGAGTTCATGAGAGATTTGGAGGAGAGCAGCTTGGAGAATGGAGTTCGCAGCTGATCAAGCAGAGTATGGGTCATAACTGCATTAGTGTATCAATGGCGTCAGTTACCACATGAAATGTTTGCGATTTTTGTTAAGTTAGAAGGAAACTAGTCCGCATAGTGATGTTTTTTTCGTCCTCCGATGACGGACGCAATTGTCTCCCGTGTCCAAAAGTTGCAGTGTCAACCATTTCTGTATAAGCAAATTAAAATAATGCATAGTCTTCAAACCTGACCATTACTAGCGACCAATTATTCAGAGCCACACAAACAACATTAACCCACTAGACTGGAAATCACTGGCTCAAGGGCCAATTCG
